# Chemical Eustress Elicits Tailored Responses and Enhances the Functional Quality of Novel Food *Perilla frutescens*

**DOI:** 10.3390/molecules24010185

**Published:** 2019-01-06

**Authors:** Youssef Rouphael, Marios C. Kyriacou, Petronia Carillo, Fabiana Pizzolongo, Raffaele Romano, Maria Isabella Sifola

**Affiliations:** 1 Department of Agricultural Sciences, University of Naples Federico II, 80055 Portici, Italy; fabiana.pizzolongo@unina.it (F.P.); raffaele.romano@unina.it (R.R.); sifola@unina.it (M.I.S.); 2 Department of Vegetable Crops, Agricultural Research Institute, Nicosia 1516, Cyprus; m.kyriacou@ari.gov.cy; 3 Department of Environmental, Biological and Pharmaceutical Sciences and Technologies, University of Campania “Luigi Vanvitelli”, 81100 Caserta, Italy; petronia.carillo@unicampania.it

**Keywords:** macro-minerals, functional food, perillaldehyde, phenolic compounds, sodium chloride, SPME-GC/MS, volatile profile

## Abstract

Consumer demand for fresh and functional horticultural products is on the rise. *Perilla frutescens*, L. Britt (*Lamiaceae*) is a potential specialty/niche crop for consumption and therapeutic uses with high contents of phenolic and volatile compounds. Plant growth, mineral composition, polyphenol profile and aroma volatile components of two perilla genotypes in response to salinity (non-salt control, 10, 20 or 30 mM NaCl) applied as chemical eustressor were assessed. Salinity suppressed growth and yield of both genotypes, although the red-pigmented genotype was less sensitive than the green-pigmented one. Mild (10 mM NaCl) and moderate (20 and 30 mM NaCl) salinity suppressed foliar potassium, magnesium, nitrate and chlorophyll a concentrations of both genotypes and increased the levels of rosmarinic acid, total polyphenols and target aroma volatile components. Green perilla showed higher yield and biomass production and higher content of protein, dry matter, calcium, magnesium, perilla ketone and *cis*-jasmone, whereas red perilla exhibited higher content of potassium, chlorophyll a, rosmarinic acid, total polyphenols, perilla aldehyde and benzaldehyde. Our findings support that chemical eustressors such as mild to moderate salinity offer valuable means to manipulate phytochemical and aroma profiles.

## 1. Introduction

Consumer interest in functional diets supporting human health and longevity has rapidly expanded worldwide and consequently demand for healthier and organoleptically appealing fresh horticultural products has increased. These changes in consumer behavior have fueled the critical reassessment of the quality of fresh horticultural products as a concept, concisely defined in a recent review as ‘a dynamic composite of physicochemical properties and evolving consumer perception, which embraces organoleptic, nutritional and bioactive components’ [1]. The bioactive components of fresh horticultural products have been the key ingredients used to generate a wide range of novel functional foods [2]. In addition, horticultural products can provide ingredients of natural-origin able to fulfill the food-preserving functions exerted by synthetic antioxidants [3]. Functional foods and natural food ingredients have thus become major lines of the global food industry, driven by increasing consumer demand, price premiums, and improving market opportunities [4]. Consequentially, growers, extension specialists and scientists have become increasingly interested in new and/or niche horticultural species rich in bioactive metabolites suitable for the production of supplements with nutraceutical, therapeutic or preservative activity [5]. 

*Perilla frutescens*, L. Britt *Lamiaceae* (formely *Labiateae*), an edible and medicinal plant used traditionally in China, Japan and other Asian countries, has recently gained wider acclaim and high value [6]. Nowadays, its cultivation in the United States, North and South Africa and Europe is widespread, with notable economic returns [7]. The seeds, leaves, and stems of this species contain vitamins, minerals, essential oils and phenolic phytochemicals valued by the food industry as flavoring and antioxidant agents for foods and drinks, and in the pharmaceutical industry as anti-allergic, anti-inflammatory and anti-oncogenic agents [5,8,9]. Perilla is present in nature in two, genetically discrete, main chemo-varietal phenotypes: the anthocyanin-rich red-pigmented cultivar known as “Zi-So”, widely grown in China, and the non-pigmented green cultivar mainly present in Japan and known as “Shisoyo” or “Shiso” [10,11]. Red perilla is mainly used for its antioxidant activity and as a food colorant [12], while the green phenotype is mainly used for food, skin cream, and medicines for allergic dermatitis [13]. Chinese and Japanese restaurants widely use Zi-So or Shiso leaves to accompany various fish dishes, in particular with Sa-Shi-Mi (fresh fish slices), not only as a fragrant condiment but also because of its bioactive properties that fence food against microbial contamination [14]. The main active phytochemicals of perilla are perillaldehyde, an essential oil which imparts the unique and characteristic flavor to the plant leaves, and rosmarinic acid, a phenolic acid used for culinary and clinical applications [15,16]. 

Perilla usually grows in wild areas or is cultured outdoors under growing conditions and practices that do not guarantee standardized quality and bioactive content, which may additionally vary in relation to the genetic material used (e.g., green vs. red perilla) [1,16]. Given the important properties of this plant and its high economic value, extensive research has been conducted concerning the effects of crop nutrition and environmental conditions on its metabolite profile [17]. However, few scientific papers have described the positive effects of environmental factors on crop performance and especially on the quality traits of this valuable novel crop. In fact, in studies of the modulation of phenolic phytochemicals in both red and green perilla grown under field conditions and harvested in different seasons and years [5,15], or grown in controlled conditions under 100–300 µE PPFD combined with 1–3 dS·m^−1^ of nutrient solution electrical conductivity [16], only the unfavorable effects of the studied environmental factors were considered. On the contrary, recent studies on the effect of combined or milder environmental stresses on plants have shown tailored responses ascribable to the stress-related plasticity of plants which can strategically command metabolic reshuffling and accumulation of bioactive compounds in order to cope with stress-induced sub-optimal conditions [1,18,19,20]. In particular, positive stress administered at low intensity, also known as eustress, can trigger the biosynthesis and accumulation of bioactive secondary compounds (carotenoids, phenolic compounds, organosulfuric compounds, polyamines etc.) which enhance the functional properties and the nutritional value of the final product [21,22]. Stress elicits dose-dependent responses, or as stated by Paracelsus ‘dose makes the poison’. Among the known types of eustress, salinity is one able to improve the quality of horticultural products provided an optimal, mild dose is used, in compliance to the famous sectiodivina. Recently, Petropoulos et al. [23] found that moderate salinity levels (up to 6.0 dS·m^−1^) increased the ascorbic acid and α-tocopherol contents and the antioxidant activity of the important leafy vegetable *Cichorium spinosum*. Beneficial effects of mild salt-stress (<30 mM NaCl) were also demonstrated in artichoke and cardoon leaves, which showed an increase of total phenolics, chlorogenic acid, cynarin and luteolin contents and antioxidant activity [24]. However, since response to salt-stress is a cultivar-dependent feature, the choice of proper genotype is crucial for achieving high phytochemical concentrations using salinity eustress [21,22]. 

To our knowledge, nothing is known regarding the ability of salinity as a eustress factor or as a chemical eustressor to modulate leaf secondary metabolites and aroma volatiles, nor regarding the salinity-genotype interaction in perilla species as a means for improving leaf quality. With respect to the above considerations, our aim was to assess the effect of mild to moderate salinity levels applied in the nutrient solution on productivity, colorimetry, mineral composition, protein content, polyphenol profile and aroma volatile components of red and green-pigmented perilla genotypes grown in soilless culture.

## 2. Results and Discussion

### 2.1. Fresh Yield, Growth Response and Colorimetric Attributes

Plant final height and leaf area were influenced by salinity and genotype with no significant salinity × genotype interaction, whereas the leaf number, leaf fresh yield, as well as dry shoot biomass were affected by salinity × genotype interaction (Table 1). The significant depression of plant growth parameters (leaf number and area, leaf fresh yield and dry biomass) with increasing salinity level in the nutrient solution has been demonstrated in previous greenhouse studies on cardoon/artichoke and spiny chicory [23,24]. Intra-specific cultivars/genotypes may develop different salt-tolerance mechanisms depending on the severity of stress in terms of concentration and time of exposure [21,22]. This was the case in the current study since the fresh yield and dry biomass of both genotypes decreased with increasing salinization, although the red-pigmented genotype was less affected than green-pigmented one at 20 and 30 mM NaCl. Specifically, the percentage of fresh yield reduction in comparison to non-salinized control plants was 7.8%, 16.5% and 48.6% in red perilla and 22.0%, 44.4% and 78.0% in green perilla at 10, 20 and 30 mM NaCl concentration in the nutrient solution, respectively (Table 1). This differential genotype response was also confirmed for dry shoot biomass and leaf number. The higher sensitivity of green perilla to salinity is attributed to the higher sodium concentrations in leaf tissue compared to red perilla (Table 2). Averaged over salt treatment levels, red perilla was able to accumulate 48.5% less sodium and to maintain potassium-to-sodium ratio (salt tolerance indicator) two-fold higher than the green cultivar (Table 2). The improved crop performance of red perilla in terms of fresh yield and dry biomass at the three levels of salinity stress applied could be associated to decreased stomatal resistance, which enhances the net CO_2_ assimilation rate (data not shown), and consequently plant vegetative growth and productivity. Moreover, it is worth noting that the tested genotypes demonstrated marked differences in their fresh yield and dry biomass potential. Under non-saline conditions, the fresh and dry yield of green perilla was respectively 43% and 68% higher than that of red perilla (Table 1). This is in agreement with previous research which demonstrated that green perilla cultivars generally grow faster than red ones under both controlled and open-field conditions [16,25]. 

Among the physical properties of leafy vegetables that strongly influence consumer preference, acceptability and choice making is visual appearance, in particular leaf color [26,27]. The coloring pigments (i.e., carotenoids, phenols, anthocyanins and betalains) may also be considered as an indicator for estimating the antioxidant properties of edible and medicinal plants, where red and dark green colored leafy vegetables are richer in nutrient content than lighter colored vegetables [28]. This was the case in the present study where genotype was expectedly the determining factor affecting Perilla leaf color, given that cultivars of distinct colorimetry were examined. The highest redness values recorded in red perilla were expectable since this chemotype is characterized by high presence of specific pigments (phenols and anthocyanins) involved in leaf coloration. Less significant was the effect of salinity on leaf brightness (L*), which overall increased in response to increasing NaCl concentration in the nutrient solution (Table 1). The predominance of the genotypic effect completely masked the effects of salinity on color components a* and b*. However, a closer look at color component a* shows that in the green cultivar it dropped in response to the 30 mM NaCl treatment, denoting loss of greenness and a transition towards the achromatic center of the CIELAB chromatic sphere [29]. A one-way analysis of variance (data not presented) within cultivars confirmed the significance of this effect on the more salt-sensitive green cultivar.

### 2.2. Dry Matter, Protein Content and Mineral Profile

The leaf dry matter (DM) content recorded in the green and red perilla plants ranged from 18.3% to 22.4% and from 16.1% to 21.7%, respectively (Table 2). Salt treatment increased the leaf DM percentage with the highest values observed in green perilla at 20 and 30 mM as well as in red perilla plants at 30 mM NaCl (Table 2). Analysis of variance also highlighted that the genotype × salinity interaction was significant for proteins with the highest values observed in green and red perilla under non-saline conditions (Table 2). Our findings on the proteins content (237–282 and 204–274 g·kg^−1^ dw in green and red perilla, respectively) were proximate to the 130 and 206 g·kg^−1^ dw reported by Peiretti [30] on *Perilla frutescens* harvested at three morphological stages (42, 49 and 52 days after sowing).

It is well known that the presence of major minerals (P, S, K, Ca and Mg) in the human diet is crucial since these macronutrients have multiple properties and functionality for human metabolism and homeostasis [31]. In fact, Levander [32] stated that fruits and vegetables normally contribute 11%, 35%, 7% and 24% of the total human dietary intake of phosphorus, potassium, calcium and magnesium, respectively. Among the macro cations and anions analyzed, K+ was the main mineral constituent followed by PO_4_^3−^, Ca^2+^, Mg^2+^ and finally SO_4_^2−^ (Table 2). To our knowledge, this is the first scientific work reporting the leaf mineral profile of green and red perilla plants, which could constitute an important information for the food composition databases.

Concerning the influence of genetic material on mineral composition, significant variation was recorded for K^+^, Ca^2+^, Mg^2+^ and Na^+^, whereas neither genotype nor salinity treatment had a significant effect on SO_4_^2−^ concentration in leaves (avg. 0.27 g·kg^−1^ dw; Table 2). The highest values of calcium, magnesium and sodium were recorded in green perilla, whereas an opposite trend was observed for potassium with the highest concentration observed in red perilla (Table 2). Nutritional interest in vegetables such as perilla containing high concentrations of minerals (K^+^ in red perilla, Ca^2+^ and Mg^2+^ in green perilla) has been associated with the health-promoting functions of these macro-minerals, such as lowering of blood pressure and waste elimination (for K), enhancement of skeletal health as well as reduced incidence of osteoporosis (for Ca and Mg) [31]. The PO_4_^3−^, K^+^, Ca^2+^ and Mg^2+^ concentrations in both green and red perilla leaves were negatively influenced by salt stress treatment but only at 20 and 30 mM NaCl (Table 2). The nutritional disorder incurred by perilla plants at 20 and 30 mM NaCl has been associated to several mechanisms, including: (i) osmotic effects of Na^+^ and Cl^−^; (ii) reduction in cell membrane integrity (i.e., permeability); and (iii) alteration in the uptake and translocation of monovalent (K^+^) and bivalent cations (Ca^2+^ and Mg^2+^) due to the competitive interactions with Na^+^ [33]. Therefore, green and to a much lesser degree red perilla plants become more sensitive to Na^+^ and/or Cl^−^ injuries (especially with 20 and 30 mM NaCl), resulting in biomass reduction and fresh yield loss 

### 2.3. Nitrate and Chlorophyll a Contents, Phenolic Composition and Total Phenolic Content

From a botanical point of view, vegetable crops belonging to the family of *Lamiaceae* (i.e., basil, thyme) are classified as high nitrate accumulating species (2500–5000 mg·kg^−1^ fw [34]. This was the case in the current study, since the nitrate contents of green and red perilla under non-saline control were 2825 and 2780 mg·kg^−1^ fw, respectively, with no significant differences between the two perilla genotypes (Table 3). Interestingly, the addition of NaCl in the nutrient solution could be considered a useful tool for mitigating the high accumulation of anti-nutrients (nitrate) in both perilla cultivars, due to the well-known antagonism effect between these two monovalent anions (NO_3_^−^ and Cl^−^). In our experiment, concentrations of both sodium and chloride in the root zone decreased the nitrate uptake, translocation and accumulation in perilla leaves by 34%. A putative mechanism behind the decrease in nitrate concentration could be the reduced plant growth rate and development, leading to a down regulation of net nitrate uptake through a lower request for nitrogen [35,36]. 

Significant genotype variation was demonstrated in terms of perilla pigmentation expressed in total chlorophyll a concentration (Table 3). When averaged over salinity treatments the highest chlorophyll a concentration was recorded in red compared to green perilla leaves (Table 3).

Sodium and chloride concentrations in the nutrient solution interfere with multiple aspects of plant biochemistry/physiology including chlorophyll biosynthesis. In the current experiment, salinity-induced degradation of chlorophyll as well as inhibition of its synthesis [37], presumably a consequence of Na^+^ induced Mg^2+^ deficiency, has been driven by an extreme ratio of Na^+^/Mg^2+^ [38].

Nutritional and functional properties of perilla leaves are associated with the high concentration of secondary metabolites, in particular polyphenols [1,22]. Moreover, the quantitative and qualitative variations of bioactive molecules (phenolic acids) depend on several preharvest factors, including species/genotypes, environmental conditions, developmental and physiological stage as well as the biotic and abiotic elicitors [22,38]. The major phenolic compound recorded in both green and red perilla leaves was rosmarinic acid, a caffeic acid derivate (synthesized through the phenylpropanoid pathway) representing more than 93% of the total target polyphenols determined by HPLC-DAD (Table 3). Increasing the nutrient solution salinity from 1 to 30 mM NaCl enhanced the functional quality of leaves by increasing rosmarinic acid and total polyphenol levels (Table 3). Similar beneficial effects of salinity (30 mM NaCl) on total phenolic compounds and major polyphenols (chlorogenic acid, cynarin and luteolin) of artichoke and cardoon leaves were reported previously by Colla and co-workers [24]. Elicitation effect of salinity on rosmarinic acid is very important, particularly in light of clinical and pre-clinical studies that have attributed to rosmarinic acid present in Lamiaceae species, such as perilla, widely ranging therapeutic and health-promoting (anticancer, neuroprotective, antiatherogenic, antibacterial, antiviral and antidiabetic) properties [39,40,41,42]. The application of 10 mM NaCl to the nutrient solution elicited an increase in total polyphenols synthesis and accumulation in both green and red perilla compared to the non-saline control treatment, thus contributing added value to this product for human diet. Further increase of the nutrient solution salinity (20 and especially 30 mM NaCl) however had a negative effect on total phenolics content (Table 3). A putative mechanism involved in the decrease of total polyphenols at 20 and especially 30 mM NaCl is that the antioxidant system of red and green perilla did not efficiently support reactive oxygen species (ROS) scavenging and cellular water homeostasis [22] as demonstrated by the sharp decrease in plant growth parameters and crop productivity (especially in green perilla). In addition to the positive effect of a chemical eustressor such as salinity, enhancing the production of polyphenols (rosmarinic and caffeic acids and flavonoids) by chemical elicitors such as chitosan, jasmonic acid and its ester, methyl jasmonate has been previously demonstrated effectively by several authors on culinary, medicinal and aromatic plants belonging to the family of Lamiaceae (e.g., *Ocimumbasilicum* and *Salvia officinalis*; [43,44,45]).

Regarding the effect of perilla genotypes, the content of sinapic acid as well as total polyphenols were 10 and 1.5-fold higher respectively in red than green perilla (Table 3). The strong effect of genetic material on target and total polyphenols has been previously demonstrated in other vegetable species such as potato [46], tomato [47], artichoke [48,49] and garlic [50]. The marked differences found between red and green perilla in terms of phenolics suggest the possibility of exploiting this quantitative variability in breeding programs aiming to select specific genotypes with fortified phytonutrient content.

### 2.4. Leaf Aroma Volatile Profile

It is well established that volatile profiles in Lamiaceae may change in relation to several interacting preharvest factors, including genotype, chemotype, cultural environment, agronomic practice, geographic location, as well as chemical elicitation agents such as jasmonic acid and chitosan [45,51,52,53]. This was the case in the current experiment, since the two perilla genotypes differed widely in the abundance of the two major aroma volatile components identified, perillaldehyde and perilla ketone (Table 4). Perillaldehyde (PA) constituted 41.6% of the total volatile profile in red perilla while it was not detected in the green one; on the other hand, perilla ketone (PK) was the most abundant (51.5%) volatile component detected in green perilla, whereas it was non-detectable in red-pigmented perilla (Table 4). In addition to PA and PK, benzaldehyde (26.7%) and cis-jasmone (21.2%) were the second most abundant compounds in red- and green-pigmented perilla, respectively (Table 4). The remaining major compounds such as benzaldehyde and β-linalool detected in both cultivars were 12.7- and 1.5-fold higher in red compared to green perilla, respectively whereas an opposite trend was recorded for caryophyllene (Table 4). Durenol and thymoquinone were only detected in red perilla leaves, whereas perillene and 4-trifluoroacetylimidazole were exclusively recorded in green cultivar (Table 4). 

The chemotype profiling revealed that *Perilla frutescens* var. *frutescens* produced exclusively perilla ketone (PK) and was classified as PK-chemotype, whereas *Perilla frutescens* var. *crispa* contained perillaldehyde (PA) instead of PK and was classified as PA-chemotype. These findings are in agreement with those of Martinetti et al. [25] who reported that open-field Japanese red and green *Perilla frutescens* var. *crispa* cultivars (‘AoShiso’, ‘Aka Shiso’, ‘Qing Su’ and ‘Purple Zi Su’) produced exclusively PA, whereas Korean green perilla (*Perilla frutescens* var. *frutescens*) contained PK. The high abundance of PA in red perilla adds nutritional value to the crop since this is the key volatile component responsible for the premium aroma and taste of perilla [15]. On the other hand, PK a terpenoid component present in green perilla leaves has been demonstrated to be toxic to animals (cattle and horses) causing pulmonary edema [9], but the effect as well as the toxic dose to humans is still equivocal [25]. Interestingly, several studies [54,55] demonstrated that PA and PK trigger the Transient Receptor Potential (TRPA1) cation channels which are actively implicated in several biological mechanisms such as the chemesthetic and trigeminal sensations, indicating perilla as a potent functional vegetable. 

Upon salinization, the relative abundance of major aroma volatiles remained similar (no significant difference) to that of non-stressed plants, in both red- and green-pigmented perilla, with the exception of 1-octen-3-ol, perilla ketone, cis-jasmone and 4-trifluoroacetylimidazole (Table 4). Irrespective of perilla genotypes, treatment with 20 mM NaCl resulted in an increase in the levels of perilla ketone and 4-trifluoroacetylimidazole (Table 4). Moreover, a significant increase in the contents of 1-octen-3-ol (with 20 and 30 mM NaCl) and cis-jasmone (in all salt-stress treatments) was observed compared to untreated plants (Table 4). Triggering the biosynthesis and accumulation of cis-jasmone could be of high interest to the cosmetic industry since this volatile component is considered an important aroma agent for perfume, fragrance as well as in aromatherapy. The increase in essential oils content and their composition under salt stress conditions has been reported previously on several species belonging to the Lamiaceae family such as Satureja hortensis, Salvia officinalis, Thymus vulgaris, Ocimum basilicum and Salvia mirzayanii [56,57,58,59,60]. In contrast, other studies have shown reduction or no difference in the pooled content of essential oils in lemon balm, sweet marjoram as well as basil in response to salt stress [61,62]. Conflicting reports concerning the responses of Lamiaceae species to salinity and the modulation of leaf volatile composition could be interpreted in the context of species-specific effects that may additionally depend on the magnitude of the applied stress, i.e., time of exposure and salt concentration in the nutrient solution [61].

### 2.5. Principal Component Analysis

**Figure 1:**
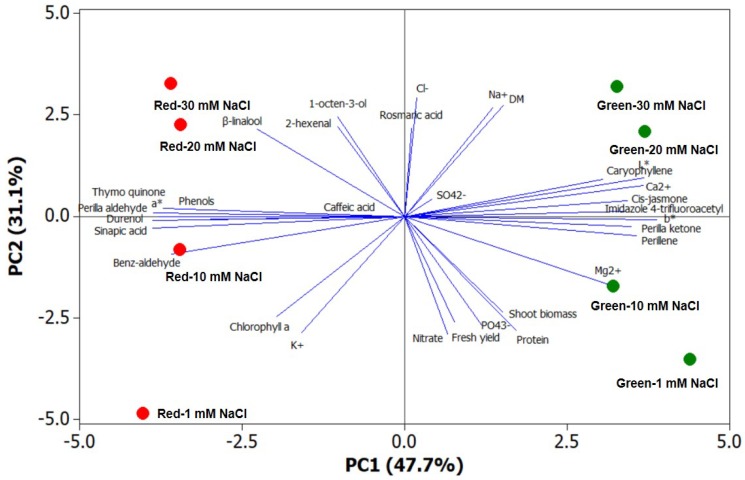
Principal component loading plot and scores of principal component analysis (PCA) of colorimetric data (L*, a*, b*), fresh yield, dry biomass, dry matter content, mineral concentrations (NO_3_^−^, N, PO_4_^3−^, K^+^, SO_4_^2−^, Ca^2+^, Mg^2+^, Na^+^, Cl^−^), protein and chlorophyll a contents, phenolic composition (rosmarinic, caffeic and sinapic acids), total phenolic content and aroma volatile compounds in green and red perilla grown under increasing NaCl concentration in the nutrient solution (1, 10, 20 or 30 mM NaCl).

A comprehensive view of the phytochemical composition as well as the aroma volatile profile of the two perilla chemotypes in response to mild-salinity as chemical eustressor was obtained through principal component analysis (PCA). The first three principal components (PCs) were associated with eigenvalues higher than 1 and explained 87.1% of the cumulative variance, with PC1 accounting for 47.7%, PC2 for 31.2% and PC3 for 8.2% (Table 5). PC1 was positively correlated with visual appearance traits (L* and b*), the two bivalent cations (Ca^2+^ and Mg^2+^), and several volatile compounds (perillene, caryophyllene, perilla ketone, *cis*-jasmone, 4-trifluoroacetylimidazole and thymoquinone); it was negatively and strongly correlated to sinapic acid, total polyphenols, benz-aldehyde, perilla aldehyde and durenol (Table 5). PC2 was positively correlated to fresh yield, dry biomass, protein, nitrate and chlorophyll a contents and negatively associated with rosmarinic acid, 2-hexenal and 1-octen-3-ol. Moreover, the loading matrix indicates the relations among the examined qualitative variables, based on which the two vectors with an angle lower than 90° are positively correlated, while an angle higher than 90° means that these variables are negatively correlated. In the current study, variation in Na^+^ concentration was most closely aligned with leaf dry matter percentage, and variation in total polyphenols was strongly correlated to perilla aldehyde (Figure 1).

The efficacy of PCA for plotting species/cultivars characterization as well as its interpretive usefulness in preharvest and postharvest studies on multiple quality traits has been reported previously in a series of scientific papers [38,63,64]. In the current study, the score plot of the PCA superimposed on the above matrix of variables revealed strong clustering of the two perilla genotypes grown under mild to moderate salt stress conditions, with green-pigmented perilla concentrating plant growth parameters, most of the mineral composition (except the K^+^) and leaf colorimetry, while the red cultivar stands out for target and total polyphenols as well as several major aroma volatile components (Figure 1). Particularly, the green-pigmented perilla was positioned on the positive side of PC1 in the upper and lower right quadrants of the PCA score plot. The upper right quadrant included green cultivar treated with 20 and 30 mM NaCl that delivered leaves with high concentration of toxic ions (Na^+^ and Cl^−^) as well as high dry matter percentage. Green perilla grown under non-saline solution or treated with 10 mM NaCl were positioned in the lower right quadrant, characterized overall by higher plant growth parameters (leaf fresh yield and shoot dry biomass), protein and Mg^2+^ concentration (Figure 1). 

The upper left quadrant depicted treatments (red perilla treated with 20 and 30 mM NaCl) characterized by high levels of 1-octen-3-ol, 2-hexenal and β-linalool. Finally, treatments in the lower left quadrant (red perilla at 1 mM and especially 10 mM NaCl) had the highest sinapic acid, total polyphenols and key volatile compounds such as perilla aldehyde (Figure 1). Altogether, the PCA outputs provide an important piece of information, namely that the targeted selection of perilla cultivar along with the application of a chemical eustressor, such as mild salt stress, could enhance the nutritional and functional quality of perilla grown in controlled environments.

## 3. Materials and Methods

### 3.1. Greenhouse Conditions and Plant Material

A greenhouse experiment was carried out during the spring-summer growing season to examine the effect of four sodium chloride (NaCl) concentrations in the nutrient solution on the growth, bioactive composition and aroma volatile profile of green perilla (*Perilla frutescens* var. *frutescens*; CN Seeds Ltd., Cambridgeshire, UK) and red perilla (*Perilla frutescens* var. *crispa*; CN seeds Ltd.). Seeds of both green and red perilla were germinated in vermiculite on 13 April 2015. Green and red perilla seedlings were transplanted 23 days after sowing (5 May), at the two-true-leaf phenological stage, into plastic black pots (20 cm diameter) containing 5.3 L of a peat/perlite in 2:1 volume ratio. Plastic pots were arranged in double rows 90 cm apart, and the space between plants within a row was 30 cm. The distance between the centers of double rows was 222 cm, resulting in a plant density of 3 plants·m^−2^. The trial was performed at the experimental station of the Department of Agricultural Sciences, University of Naples Federico II, Bellizzi, south Italy (43°31 N, 14°58 E; 60 m above sea level). Perilla plants were cultivated under natural light conditions and the daily air temperature inside the glasshouse was maintained between 18 and 32 °C.

### 3.2. Experimental Design, Sodium Chloride Application and Nutrient Solution Management

Eight treatments derived by a factorial combination of four NaCl concentrations in the nutrient solution (non salt control 1, 10, 20 or 30 mM) and two perilla cultivars (green and red) were delivered. The treatments were arranged in a randomized complete-block design with three replicates per treatment, amounting to a total of 24 experimental units (i.e., plots). Each experimental units (replicates) consisted of 8 plants for a total number of green and red perilla of 192 plants. The first and last plant of each experimental were considered as guards, and the biometric and chemical composition measurements were carried out on each of the six plants per experimental unit separately.

The basic nutrient solution was a modified Hoagland formulation with a mmol·L^−1^ composition of: 14.2 N-NO_3_^−^, 1.8 S, 1.0 P, 0.6 N-NH_4_^+^, 6.5 K, 4.0 Ca, 2.2 Mg, 1 Na, and 1 Cl and (in μmol·L^−1^) 20 Fe, 10 Mn, 0.4 Cu, 1.5 Zn, 20 B, and 0.3 Mo, with an electrical conductivity (EC) of 2.0 dS·m^−1^. The three saline nutrient solutions had the same basic nutrient solution plus an additional 9, 19 and 29 mM NaCl, resulting in an EC of 3.0, 3.9 and 5.1 dS·m^−1^, respectively. The pH of the four nutrient solutions was 6.0 ± 0.3. All nutrient solutions were prepared using deionized water. Saline treatment started on 12 May, eight days after transplanting (DAT).

The four nutrient solutions (control and three saline treatments) were pumped from independent 150 L tanks and delivered through a drip irrigation system through one emitter per plant at a rate of 2 L·h^−1^. Green and red perilla plants received the same solution volume, allowing for a leaching fraction of 20% to avoid any build-up of salinity in the substrate, thus maintaining the EC in the growing media at a level proximate to that of the treatment nutrient solution.

### 3.3. Yield and Growth Measurements, Collection of Samples

At the end of the greenhouse experiment (52 DAT), the final plant height as well as the number of leaves per plant were recorded on six green and red plants per replicate. The total leaf area per plant was measured using an electronic area meter (Li-Cor3000, Li-Cor, Lincoln, NE, USA). Six plants of green and red perilla per experimental unit were harvested and separated in leaves and stems. Both leaf and stem tissues were dried at 65 °C for 72 h until they reached a constant weight, recorded on an analytical balance (Denver Instruments, Denver, CO, USA), which corresponded to their dry biomasses. Shoot dry biomass was equal to the sum of the aerial vegetative parts (leaves + stems). Part of the dried leaf tissue was used for mineral composition. For the identification and quantification of phenolic compounds by HPLC and also for leaf volatile profiling by HS-SPME and GC/MS, fresh leaf samples from two plants per experimental plot were instantly frozen in liquid nitrogen and stored at −80 °C before lyophilized them in a Christ, Alpha 1-4 (Osterode, Germany) freeze drier.

### 3.4. Leaf Color Measurements

Before harvest, leaf color was measured on the upper part of ten leaves per experimental unit plot, using an 8 mm-aperture Minolta CR-300 Chroma Meter (Minolta Camera Co., Ltd. Osaka, Japan). The Chroma meter was calibrated with a Minolta standard white plate before sampling green and red perilla leaves. The Commission internationale de l’éclairage (CIE) color space parameters recorded in the present study were lightness (L*) and chroma components a* and b*. L* (ranging from 0 = black to 100 = white), a* (ranging from green [−60] to red [+60]), b* (ranging from blue [−60] to yellow [+60]), and readings were transformed to those of the L, a, b color space.

### 3.5. Protein and Mineral Profile Analysis

Dried leaf tissues ground in a Wiley Mill to pass through an 841 µm screen were used for protein and mineral content analysis. Total nitrogen (N) concentration in green and red perilla tissues was determined by the Kjeldahl method following mineralization with sulphuric acid (H_2_SO_4_, 96%, Carlo Erba Reagents, Cornaredo, Milan, Italy) in the presence of potassium sulfate and copper catalyst as described by Bremner [65] Protein content was determined following the official method 976.05 of the AOAC [66], using N-to-protein conversion factor of 6.25.

For the anions (NO_3_^−^, PO_4_^3−^ and SO_4_^2−^) and cations (K^+^, Ca^2+^, Mg^2+^ and Na^+^) leaf analysis, 0.25 g of the green or red perilla dried material were suspended in 50 mL of ultrapure water (Milli-Q, Merck Millipore, Darmstadt, Germany) and subjected to three freeze-thaw cycles in liquid nitrogen followed by 10 min shaking in a water bath (ShakeTemp SW22, Julabo, Seelbach, Germany) at 80 °C. The mixture was centrifuged at 6000 rpm for 10 min (R-10 M, Remi Elektrotechnik Limited, Valiv, India), then filtered through a 0.20 μm filter paper (Whatman International Ltd., Maidstone, UK), as described previously by Rouphael et al. [67]. Anions and cations in leaf tissues were separated and quantified by ion chromatography (ICS-3000, Dionex, Sunnyvale, CA, USA) coupled to a conductivity detector. The conductivity detector with IonPac CG12A (4 × 250 mm, Dionex) guard column and IonPac CS12A (4 × 250 mm, Dionex) analytical column were used for the cations analysis, while for the anions determination, an IonPac AG11-HC guard (4 × 50 mm) column and IonPac AS11-HC analytical column (4 × 250 mm) were employed.

### 3.6. Sample Extraction and Assay Preparation

Two hundred grams of freeze-dried leaves were extracted with 30 mL of methanol/water (70:30, *v*/*v*) by sonication at room temperature for 30 min. The mixtures were centrifuged at 8000 rpm for 5 min at room temperature, filtered through a 0.20 μm membrane (Whatman International Ltd., Maidston, UK) and then used for HPLC analysis (phenolic compounds and chlorophyll a) and total polyphenols (colorimetric) determinations.

### 3.7. Identification and Quantification of Phenolic Compounds and Chlorophyll a by HPLC-DAD

Leaf samples of green and red perilla were filtrated through a 0.20 μm membrane and analyzed by HPLC in order to quantify target phenolic compounds and chlorophyll a content, according to Kang and Lee [5]. The analysis was performed with an 1100 Series HPLC system (Agilent Technologies, Palo Alto, CA, USA) equipped with a quaternary pump (G13111A) and a diode array detector (DAD; G13114B) using a 20 µL sample injection loop. A reversed phase C18 column (150 × 4.6 mm i.d., particle size 5 μm; Agilent Eclipse XDB-C18) was employed. The eluents were 5% (*v*/*v*) formic acid in water (eluent A) and 5% (*v*/*v*) formic acid in acetonitrile (eluent B). The gradient program was as follows: 2–7% B (5 min), 7–12% B (10 min), 12–17% B (5 min), 17–25% B (5 min), 25–35% B (10 min) at a constant flow rate of 1.3 mL min^−1^. Total runtime was 35 min. Detection was performed at 320 nm for rosmarinic, caffeic and sinapic acids and at 666 nm for chlorophyll a. Identification was made by comparing retention times to rosmarinic, caffeic, sinapic and chlorophyll a standards purchased from Sigma Aldrich (Milan, Italy), by spiking samples with internal standards and by calculating their recovery. Calibration curves of high linearity (R^2^ > 0.999) were derived for each standard using five concentration levels (1, 10, 20, 50 and 100 mg·L^−1^). All data are presented as mean ± standard error (*n* = 3), and expressed as mg g^−1^ dry matter. In addition, as suggested by the reviewer representative HPLC-DAD chromatograms of polyphenolic (rosmarinic, caffeic and sinapic acids) and chlorophyll a extracts of *Perilla* leaves monitored at 320 nm and 666 nm, respectively are shown in Figure S1. 

### 3.8. Determination of Total Phenolics

Total phenolics content was determined using the Folin-Ciocalteu colorimetric method with gallic acid as a standard as previously described [68]. Green and red perilla extracts (100 μL) were mixed with 0.2 mL Folin-Ciocalteu reagent and 2 mL of distilled water, and incubated at room temperature for 3 min. Total polyphenols were assayed after adding 1 mL of 20% sodium carbonate to the mixture and incubating for 1 h at room temperature. The absorbance of the resulting blue color was measured at 765 nm with a UV-1601PC UV-Visible scanning spectrophotometer (Shimadzu, Milan, Italy). Quantification was done with respect to the standard curve of gallic acid, and the result was expressed as mg gallic acid equivalents per g dry weight. Total polyphenols analysis was performed in triplicate.

### 3.9. SPME-GC/MS of Volatile Compounds

SPME-GC/MS was used for sampling and analysis of the volatile compounds in powdered green and red perilla leaves, according to Huang et al. [69] with slight modifications. A SPME holder containing a fused-silica fibre coated with a 50/30 µm layer of divinyl-benzene/carboxen/polydimethylsiloxane (DVB/CAR/PDMS; Supelco, Bellefonte, PA, USA) was used for the absorption of the volatile compounds. Two grams of freeze-dried powdered leaves were weighed and immediately introduced into a 20-mL headspace vial. The SPME fibre was exposed in the head space of the vial for 20 min at 45 °C and then introduced directly into the GC injector where the thermal desorption of the analytes was performed at 250 °C for 5 min. An Agilent 6890N GC system equipped with a 5973 mass detector (Agilent Technologies, Santa Clara, CA, USA) were used.

The analytes were separated on a 30 m × 0.250 mm capillary column coated with a 0.25 µm film of 5% diphenyl l95% dimethylpolysiloxane (HP5MS J&W Scientific, Folsom, CA, USA) and were inserted directly into the ion source of the mass detector. Splitless injection was used for the samples. The column oven temperature was ramped at 10 °C min^−1^ from an initial temperature of 50 (held for 2 min) to 150 °C, then at 15 °C·min^−1^ to 300 °C, which was held for 10 min. The injection and ion source temperatures were 250 and 230 °C, respectively. Helium (99.999%) was used as carrier gas at a flow rate of 1 mL min^−1^. The ionizing electron energy was 70 eV and the mass range scanned was 40–450 amu in full-scan acquisition mode. The volatile compounds were identified by comparing their retention indices and mass spectra with those found in the libraries NIST Atomic Spectra Database version 1.6 using a range of 85–100% similarity values. The relative amounts were calculated on the basis of peak-area ratios. Analyses of volatile compounds were also performed in triplicate. Representative SPME-GC/MS chromatogram of aroma volatile compounds of perilla leaves is reported in Figure S2.

### 3.10. Statistical Analysis of Data

Analysis of variance (two-way ANOVA) of the experimental data was performed using IBM SPSS Statistics 20 for Windows (SPSS Inc., Chicago, IL, USA). To separate treatment means within each measured parameter, Duncan’s Multiple Range Test (DMRT) was performed at *p* ≤ 0.05. Principal component analysis (PCA) was also conducted using Minitab 16.2.1 statistical software, aimed to extract trends when multiple qualitative variables were used by formulating new variables correlated to the original ones. Since the variables have different units of measurement, therefore those variables were standardized before conducting the PCA. The PCA outputs included treatment component scores as well as variable loading to each selected component.

## 4. Conclusions

Genotypic variation in novel specialty crops, such as perilla along with the effective application of mild to moderate salinity eustress aimed at enhancing the bioactive content and secondary metabolites of vegetable crops constitute upcoming research areas attracting the interest of growers, consumers, nutritionists as well as food researchers. Greenhouse perilla plant performance and nutritional, functional quality and leaf aroma profiles were assessed in a bifactorial approach accounting for the effects of increasing NaCl concentration in the nutrient solution and genotype. Our results indicate that fresh yield, biomass production, mineral composition, target and total polyphenols and also aroma volatile composition were strongly influenced by genotype with the red-pigmented genotype exhibiting higher levels of K, chlorophyll a, sinapic acid, total polyphenols and select aroma components (benzaldehyde and β-linalool). Our findings also demonstrated that increasing salinity in the nutrient solution suppressed plant growth parameters, especially in the green-pigmented perilla. However, the application of mild-stress (10 mM NaCl) improved functional quality aspects such as polyphenols and decreased the levels of anti-nutrients such as nitrate, whereas key aroma compounds (especially in green perilla) were improved by the application of 30 and especially 20 mM NaCl in the nutrient solution. Plant growth and the biosynthesis of bioactive molecules may be optimized according to a two-stage strategy [70,71] in the application of salinity as a chemical eustressor: during the initial phenological stages plants are grown under optimal conditions, then secondary metabolism is triggered through eustress-mediated elicitation of the biosynthesis and accumulation of desired bioactive molecules.

## Figures and Tables

**Table 1:** Analysis of variance and mean comparisons for plant height, number of leaves per plant, total leaf area, leaf fresh weight, shoot dry biomass, and leaf colour: L* (brightness), a* (+a* = red; −a* = green) and b* (+b* = yellow; −b = blue) in green and red perilla grown under increasing NaCl concentration in the nutrient solution.

Source of Variance	Plant Height	Leaf Number	Leaf Area	Leaf Fresh Yield	Shoot Dry Biomass	Leaf Colour		
	(cm)	(no. plant^−1^)	(cm^2^·plant^−1^)	(g·plant^−1^)	(g·plant^−1^)	L*	a*	b*
Genotype (G)	*	ns	*	ns	**	***	***	***
Salinity (S)	**	***	***	***	***	*	ns	ns
C × S	ns	**	ns	*	**	ns	ns	ns
Genotype								
Green	44.2 ± 2.2 a	115.0 ± 13.9 a	1984.7 ± 288.1 b	50.3 ± 10.1 a	15.8 ± 1.7 a	36.0 ± 0.7 a	−6.7 ± 0.5 b	15.2 ± 0.6 a
Red	39.2 ± 1.3 b	121.1 ± 6.5 a	2422.3 ± 182.2 a	45.2 ± 4.5 a	11.8 ± 0.7 b	25.9 ± 0.7 b	4.5 ± 0.3 a	0.9 ± 0.5 b
Salinity (mM NaCl)								
1	47.4 ± 2.6 a	164.2 ± 8.7 a	3216.2 ± 174.3 a	67.0 ± 9.5 a	18.9 ± 1.9 a	28.9 ± 2.7 c	−1.0 ± 2.8 a	8.3 ± 3.6 a
10	43.8 ± 2.4 ab	132.7 ± 6.7 b	2564.5 ± 132.2 b	56.3 ± 6.6 ab	15.2 ± 1.0 b	29.9 ± 2.4 bc	−1.9 ± 2.6 a	8.1 ± 3.1 a
20	40.9 ± 1.8 b	105.9 ± 10.1 c	1983.0 ± 260.0 c	45.0 ± 8.2 b	13.0 ± 1.1 b	32.9 ± 2.3 a	−1.6 ± 2.6 a	9.3 ± 3.3 a
30	34.7 ± 1.7 c	69.4 ± 8.9 d	1050.1 ± 221.0 d	22.8 ± 5.6 c	7.0 ± 1.2 c	32.1 ± 2.0 ab	0.1 ± 2.3 a	6.5 ± 3.1 a
G × S								
Green-1 mM NaCl	52.2 ± 3.4 a	184.2 ± 8.7 a	3269.1 ± 332.3 a	78.8 ± 8.4 a	23.7 ± 1.2 a	34.9 ± 0.7 a	−7.1 ± 0.5 a	16.3 ± 1.2 a
Green-10 mM NaCl	47.4 ± 4.0 a	132.1 ± 14. b	2421.1 ± 244.0 a	61.5 ± 10.1 ab	17.5 ± 0.8 b	35.2 ± 0.1 a	−7.6 ± 0.6 a	15.0 ± 0.3 a
Green-20 mM NaCl	41.0 ± 2.4 a	93.2 ± 13.5 c	1648.1 ± 396.2 a	43.8 ± 15.0 bc	13.2 ± 2.0 bc	37.4 ± 2.3 a	−7.3 ± 0.5 a	16.4 ± 1.9 a
Green-30 mM NaCl	36.4 ± 3.5 a	50.5 ± 1.4 d	607.0 ± 182.3 a	17.3 ± 11.7 d	6.5 ± 2.3 d	36.3 ± 1.8 a	−5.1 ± 1.4 a	13.3 ± 1.0 a
Red-1 mM NaCl	42.6 ± 2.3 a	144.2 ± 2.7 b	3163.2 ± 169.3 a	55.1 ± 2.2 b	14.1 ± 0.5 bc	22.9 ± 1.1 a	5.1 ± 0.8 a	0.2 ± 0.1 a
Red-10 mM NaCl	40.4 ± 1.2 a	133.3 ± 2.8 b	2708.0 ± 90.5 a	50.8 ± 2.5 b	12.8 ± 0.1 c	24.4 ± 0.4 a	3.8 ± 0.6 a	1.3 ± 0.8 a
Red-20 mM NaCl	40.7 ± 2.9 a	118.6 ± 13.6 bc	2317.8 ± 288.0 a	46.0 ± 9.2 bc	13.0 ± 1.3 c	28.4 ± 0.5 a	4.1 ± 0.1 a	2.3 ± 0.8 a
Red-30 mM NaCl	33.7 ± 1.8 a	88.2 ± 7.9 c	1500.2 ± 208.6 a	28.3 ± 5.9 cd	7.4 ± 1.2 d	27.9 ± 0.4 a	4.9 ± 0.5 a	−0.4 ± 1.4 a

ns, *, **, *** Nonsignificant or significant at *p* ≤ 0.05, 0.01, and 0.001, respectively. Different letters within each column indicate significant differences according to Duncan’s multiple-range test (*p* = 0.05). All data are expressed as mean ± standard error, *n* = 3.

**Table 2:** Analysis of variance and mean comparisons for mineral composition of leaves in green and red perilla grown under increasing NaCl concentration in the nutrient solution.

Source of Variance	Leaf Dry Matter (%)	Protein (g·kg^−1^ dw)	Mineral Elements (g·kg^−1^ dw)					
			PO_4_^3−^	K^+^	Ca^2+^	Mg^2+^	SO_4_^2−^	Na^+^
Genotype (G)	***	***	ns	**	***	***	ns	***
Salinity (S)	***	***	**	*	*	**	ns	***
C × S	***	*	ns	ns	ns	ns	ns	**
Genotype								
Green	20.8 ± 0.4 a	256.9 ± 5.1 a	14.0 ± 0.8 a	27.7 ± 0.6 b	12.2 ± 0.3 a	2.4 ± 0.13 a	0.3 ± 0.0 a	6.6 ± 1.4 a
Red	19.1 ± 0.6 b	234.7 ± 7.9 b	12.6 ± 0.8 a	29.0 ± 0.7 a	7.1 ± 0.3 b	1.7 ± 0.06 b	0.3 ± 0.0 a	3.4 ± 0.8 b
Salinity (mM NaCl)								
1	17.2 ± 0.4 d	277.8 ± 3.3 a	16.2 ± 1.3 a	30.3 ± 0.8 a	8.4 ± 1.3 b	2.3 ± 0.23 a	0.3 ± 0.0 a	0.2 ± 0.1 d
10	19.2 ± 0.6 c	254.6 ± 5.1 b	13.9 ± 0.6 ab	29.3 ± 1.0 a	10.0 ± 1.3 a	2.3 ± 0.22 a	0.2 ± 0.0 a	2.9 ± 0.7 c
20	21.1 ± 0.2 b	229.7 ± 7.0 c	10.6 ± 0.4 bc	27.1 ± 0.5 b	10.0 ± 0.8 a	1.9 ± 0.14 b	0.2 ± 0.0 a	7.4 ± 1.3 b
30	22.0 ± 0.3 a	220.9 ± 7.5 d	12.2 ± 0.6 c	26.5 ± 0.7 b	10.1 ± 1.3 a	1.7 ± 0.12 b	0.3 ± 0.0 a	9.4 ± 1.2 a
G × S								
Green-1 mM NaCl	18.3 ± 0.2 c	282.0 ± 2.1 a	16.8 ± 2.1 a	28.9 ± 0.6 a	11.4 ± 0.1 a	2.8 ± 0.29 a	0.3 ± 0.0 a	0.1 ± 0.0 d
Green-10 mM NaCl	20.8 ± 0.1 b	263.9 ± 6.0 b	14.7 ± 0.8 a	28.7 ± 1.9 a	12.9 ± 0.6 a	2.7 ± 0.22 a	0.3 ± 0.0 a	4.3 ± 0.8 c
Green-20 mM NaCl	21.5 ± 0.4 ab	244.4 ± 1.5 c	10.9 ± 0.5 a	26.5 ± 1.0 a	11.8 ± 0.2 a	2.2 ± 0.19 a	0.2 ± 0.1 a	10.1 ± 0.7 a
Green-30 mM NaCl	22.4 ± 0.7 a	237.1 ± 1.5 c	13.4 ± 0.8 a	26.1 ± 0.3 a	12.8 ± 0.9 a	2.0 ± 0.02 a	0.3 ± 0.0 a	11.8 ± 1.0 a
Red-1 mM NaCl	16.1 ± 0.1 d	274.0 ± 2.1 a	15.5 ± 2.0 a	31.7 ± 0.9 a	5.4 ± 0.3 a	1.9 ± 0.05 a	0.2 ± 0.0 a	0.3 ± 0.2 d
Red-10 mM NaCl	17.6 ± 0.3 c	245.3 ± 5.2 ab	13.3 ± 0.9 a	29.8 ± 1.3 a	7.0 ± 0.2 a	1.9 ± 0.15 a	0.2 ± 0.1 a	1.6 ± 0.3 d
Red-20 mM NaCl	20.7 ± 0.1 b	214.9 ± 4.0 c	10.4 ± 0.7 a	27.6 ± 0.4 a	8.3 ± 0.3 a	1.7 ± 0.05 a	0.3 ± 0.1 a	4.6 ± 1.0 c
Red-30 mM NaCl	21.7 ± 0.1 ab	204.7 ± 2.2 d	11.5 ± 0.5 a	26.8 ± 1.4 a	7.5 ± 0.5 a	1.5 ± 0.07 a	0.3 ± 0.1 a	7.1 ± 0.8 b

ns, *, **, *** Nonsignificant or significant at *p* ≤ 0.05, 0.01, and 0.001, respectively. Different letters within each column indicate significant differences according to Duncan’s multiple-range test (*p* = 0.05). All data are expressed as mean ± standard error, *n* = 3.

**Table 3:** Analysis of variance and mean comparisons for protein, nitrate and chlorophyll a contents, target phenolic acids and total polyphenols in green and red perilla grown under increasing NaCl concentration in the nutrient solution.

Source of Variance	Nitrate	Chlorophyll a	Rosmarinic Acid	Caffeic Acid	Sinapic Acid	Total Polyphenols
	(mg·kg^−1^ fw)	(mg·g^−1^ dw)	(mg·g^−1^ dw)	(mg·g^−1^ dw)	(mg·g^−1^ dw)	(mg·g^−1^ dw)
Genotype (G)	ns	**	ns	ns	***	***
Salinity (S)	**	**	*	ns	ns	***
C × S	*	ns	ns	ns	ns	**
Genotype						
Green	1937.1 ± 209.1 a	64.1 ± 5.1 b	15.7 ± 1.4 a	0.3 ± 0.0 a	0.1 ± 0.0 b	63.6 ± 3.0 b
Red	1727.6 ± 209.0 a	81.3 ± 8.2 a	15.6 ± 1.8 a	0.3 ± 0.0 a	1.2 ± 0.0 a	93.7 ± 2.0 a
Salinity (mM NaCl)						
1	2803.2 ± 205.2 a	93.3 ± 12.6 a	9.7 ± 2.0 b	0.3 ± 0.0 a	0.7 ± 0.3 a	71.8 ± 6.6 c
10	1859.7 ± 186.3 b	73.0 ± 7.1 b	18.4 ± 2.7 a	0.3 ± 0.0 a	0.7 ± 0.3 a	89.7 ± 6.0 a
20	1299.6 ± 123.4 c	65.1 ± 6.6 b	16.5 ± 0.8 a	0.3 ± 0.0 a	0.6 ± 0.2 a	82.9 ± 6.2 b
30	1366.8 ± 106.3 c	58.8 ± 6.5 b	18.1 ± 0.5 a	0.3 ± 0.0 a	0.6 ± 0.3 a	70.3 ± 8.3 c
G × S						
Green-1 mM NaCl	2780.9 ± 397.3 a	74.6 ± 0.6 a	12.1 ± 3.4 a	0.4 ± 0.0 a	0.1 ± 0.0 a	57.4 ± 2.3 f
Green-10 mM NaCl	2259.3 ± 99.3 a	71.4 ± 9.9 a	14.7 ± 3.4 a	0.3 ± 0.1 a	0.1 ± 0.0 a	76.3 ± 0.7 d
Green-20 mM NaCl	1473.0 ± 158.0 b	60.1 ± 12.4 a	17.5 ± 0.4 a	0.4 ± 0.0 a	0.1 ± 0.0 a	68.9 ± 0.6 e
Green-30 mM NaCl	1235.2 ± 52.0 b	50.4 ± 7.9 a	18.5 ± 1.0 a	0.2 ± 0.0 a	0.1 ± 0.0 aa	51.9 ± 2.7 g
Red-1 mM NaCl	2825.6 ± 229.1 a	112.0 ± 12.8 a	7.2 ± 1.6 a	0.3 ± 0.1 a	1.3 ± 0.0	86.2 ± 1.5 c
Red-10 mM NaCl	1460.1 ± 52.2 b	74.6 ± 12.4 a	22.1 ± 3.3 a	0.4 ± 0.0 a	1.2 ± 0.0 a	103.1 ± 0.7 a
Red-20 mM NaCl	1126.2 ± 145.3 b	70.1 ± 6.4 a	15.5 ± 1.5 a	0.3 ± 0.1 a	1.1 ± 0.1 a	97.0 ± 0.3 b
Red-30 mM NaCl	1498.5 ± 191.2 b	67.5 ± 4.9 a	17.4 ± 0.5	0.3 ± 0.1 a	1.1 ± 0.1 a	88.7 ± 1.0 c

ns, *, **, *** Nonsignificant or significant at *p* ≤ 0.05, 0.01, and 0.001, respectively. Different letters within each column indicate significant differences according to Duncan’s multiple-range test (*p* = 0.05). All data are expressed as mean ± standard error, *n* = 3.

**Table 4:** Analysis of variance and mean comparisons for the relative abundance of major aroma volatile components in green and red perilla grown under increasing NaCl concentration in the nutrient solution.

Source of Variance	Aldehydes			Alcohols			Terpenes		Ketones		Diazole	Quinone
	2-hexenal	Benz-aldehyde	Perilla Aldehyde	1-octen-3-ol	Durenol	β-linalool	Perillene	Caryophyllene	Perilla Ketone	Cis-jasmone	Imidazole 4-trifluoroacetyl	Thymo Quinone
Genotype (G)	ns	***	-	ns	-	*	-	*	-	-	-	-
Salinity (S)	ns	ns	ns	*	ns	ns	ns	ns	**	**	*	ns
C × S	ns	ns	-	ns	-	ns	-	ns	-	-	-	-
Genotype												
Green	5.6 ± 1.3 a	2.1 ± 0.4 b	n.d.	4.2 ± 0.6 a	n.d.	2.8 ± 0.2 b	3.5 ± 0.5	4.7 ± 0.2 a	51.5 ± 5.2	21.2 ± 3.0	4.3 ± 0.7	n.d.
Red	7.7 ± 1.6 a	26.7 ± 3.7 a	41.6 ± 2.7	5.2 ± 0.8 a	2.9 ± 0.1	4.0 ± 0.5 a	n.d.	3.6 ± 0.4 b	n.d.	n.d.	n.d.	8.2 ± 1.0
Salinity (mM NaCl)												
1	4.1 ± 1.0 a	21.3 ± 9.8 a	18.1 ± 8.4 a	3.6 ± 0.5 b	1.4 ± 0.6 a	2.6 ± 0.3 a	2.9 ± 1.4 a	4.0 ± 0.7 a	21.6 ± 9.7 b	9.0 ± 2.9 b	2.8 ± 1.4 ab	4.4 ± 2.2
10	4.4 ± 0.9 a	12.7 ± 5.5 a	25.9 ± 11.7 a	2.8 ± 0.6 b	1.5 ± 0.6 a	2.7 ± 0.2 a	1.0 ± 0.5 a	3.8 ± 0.3 a	38.7 ± 17.8 b	12.8 ± 5.8 a	1.5 ± 0.6 b	2.5 ± 0.0
20	9.8 ± 2.5 a	11.5 ± 4.3 a	19.8 ± 9.0 a	5.9 ± 1.2 a	1.4 ± 0.6 a	4.2 ± 0.7 a	1.5 ± 0.7 a	4.9 ± 0.6 a	17.9 ± 8.2 a	14.9 ± 6.7 a	3.1 ± 1.4 a	4.9 ± 2.3
30	8.2 ± 2.5 a	12.1 ± 4.8 a	19.3 ± 9.0 a	6.4 ± 1.2 a	1.3 ± 0.6	4.1 ± 0.5 a	1.7 ± 0.7 a	4.0 ± 0.3 a	24.5 ± 11.0 b	11.7 ± 5.2 a	1.8 ± 0.8 b	4.6 ± 2.1
G × S												
Green-1 mM NaCl	5.9 ± 1.5 a	2.1 ± 0.5 a	n.d.	3.8 ± 1.0 a	n.d.	2.6 ± 0.1 a	5.7 ± 1.5	5.0 ± 0.5 a	43.3 ± 1.7	25.7 ± 1.8	5.7 ± 1.6	n.d.
Green-10 mM NaCl	3.3 ± 1.1 a	1.4 ± 0.1 a	n.d.	2.0 ± 0.5 a	n.d.	2.2 ± 0.1 a	2.1 ± 0.6	3.9 ± 0.4 a	77.6 ± 9.5	18.0 ± 5.9	3.6 ± 1.3	n.d.
Green-20 mM NaCl	8.8 ± 4.9 a	3.0 ± 1.7 a	n.d.	4.5 ± 1.3 a	n.d.	3.0 ± 0.5 a	2.9 ± 0.7	5.6 ± 0.3 a	35.9 ± 3.9	29.7 ± 2.3	6.3 ± 0.5	n.d.
Green-30 mM NaCl	4.4 ± 0.1 a	1.9 ± 0.1 a	n.d.	6.4 ± 1.3 a	n.d.	3.4 ± 0.0 a	3.3 ± 0.0	4.4 ± 0.1 a	49.2 ± 0.5	23.3 ± 0.8	3.6 ± 0.1	n.d.
Red-1 mM NaCl	2.3 ± 0.5 a	40.5 ± 10.8 a	36.2 ± 5.3	3.4 ± 0.4 a	2.9 ± 0.1	2.7 ± 0.7 a	n.d.	3.0 ± 1.1 a	n.d.	n.d.	n.d.	8.7 ± 2.5
Red-10 mM NaCl	5.4 ± 1.3 a	24.0 ± 4.8 a	51.8 ± 3.9	3.5 ± 0.9 a	3.0 ± 0.1	3.3 ± 0.2 a	n.d.	3.6 ± 0.7 a	n.d.	n.d.	n.d.	5.1 ± 1.9
Red-20 mM NaCl	10.8 ± 2.4 a	20.0 ± 4.5 a	39.6 ± 3.4	7.2 ± 2.0 a	2.9 ± 0.4	5.3 ± 1.1 a	n.d.	4.1 ± 1.1 a	n.d.	n.d.	n.d.	9.9 ± 1.8
Red-30 mM NaCl	12.1 ± 4.2 a	22.4 ± 3.3 a	38.7 ± 6.1	6.4 ± 2.3 a	2.7 ± 0.2	4.8 ± 0.8 a	n.d.	3.6 ± 0.7 a	n.d.	n.d.	n.d.	9.2 ± 1.4

ns, *, **, *** Nonsignificant or significant at *p* ≤ 0.05, 0.01, and 0.001, respectively; n.d. not detected. Different letters within each column indicate significant differences according to Duncan’s multiple-range test (*p* = 0.05). All data are expressed as mean ± standard error, *n* = 3.

**Table 5:** Eigenvalues, relative and cumulative proportion of total variance, and correlation coefficients for each nutritional trait with respect to the three principal components.

Principal Components	PC1	PC2	PC3
Eigenvalue	15.3	10.0	2.6
Percentage of variance	47.7	31.2	8.2
Cumulative variance	47.7	78.9	87.1
Eigen vectors ^a^			
Fresh yield	0.208	**0.808**	0.498
Shoot dry biomass	0.383	**0.735**	0.505
DM ^b^	0.318	**−0.891**	0.034
L*	**0.941**	−0.310	0.042
a*	**−0.991**	−0.019	−0.005
b*	**0.993**	0.012	0.024
PO_4_^3−^	0.310	**0.843**	−0.133
K^+^	−0.397	**0.905**	−0.092
Ca^2+^	**0.937**	−0.248	−0.058
Mg^2+^	**0.809**	0.518	0.157
SO_4_^2−^	0.104	−0.137	0.559
Na^+^	0.340	**−0.849**	−0.336
Cl^−^	0.040	**−0.924**	−0.166
Protein	0.446	**0.881**	−0.111
Nitrate	0.177	**0.916**	0.069
Chlorophyll a	−0.498	**0.783**	−0.148
Rosmaric acid	0.024	**−0.689**	0.029
Caffeic acid	−0.120	0.001	**0.888**
Sinapic acid	**−0.993**	0.098	0.040
Phenols	**−0.872**	0.027	0.202
2-Hexenal	−0.268	**−0.701**	**0.629**
Benzaldehyde	**−0.917**	0.301	−0.130
Perilla aldehyde	**−0.969**	0.008	0.097
1-Octen-3-ol	−0.272	**−0.771**	0.067
Durenol	**−0.993**	0.038	0.074
β-Linalool	−0.586	−0.0675	0.283
Perillene	**0.914**	0.139	0.159
Caryophyllene	**0.777**	−0.302	0.347
Perilla ketone	**0.892**	0.071	−0.169
*cis*-Jasmone	**0.876**	−0.134	0.024
4-trifluoroacetylimidazole	**0.868**	−0.053	0.164
Thymoquinone	**−0.952**	−0.055	0.127

^a^ Boldface factor loadings are considered highly weighed; ^b^ DM, dry matter.

## Supplementary Materials

Supplementary Materials are available online. Figure S1. Representative HPLC-DAD chromatogram of polyphenolic (rosmarinic, caffeic and sinapic acids) and chlorophyll a extracts of perilla leaves monitored at 320 nm and 666 nm, respectively; Figure S2. Representative SPME-GC/MS chromatogram of aroma volatile compounds of perilla leaves.
