# Supplementary material for: Chemical Eustress Elicits Tailored Responses and Enhances the Functional Quality of Novel Food Perilla frutescens
Source: Molecules. 2019 Jan 6;24(1):185. doi: 10.3390/molecules24010185 (PMC6337370; doi:10.3390/molecules24010185)
Supplement: Supplementary file 1 [file molecules-24-00185-s001.pdf]

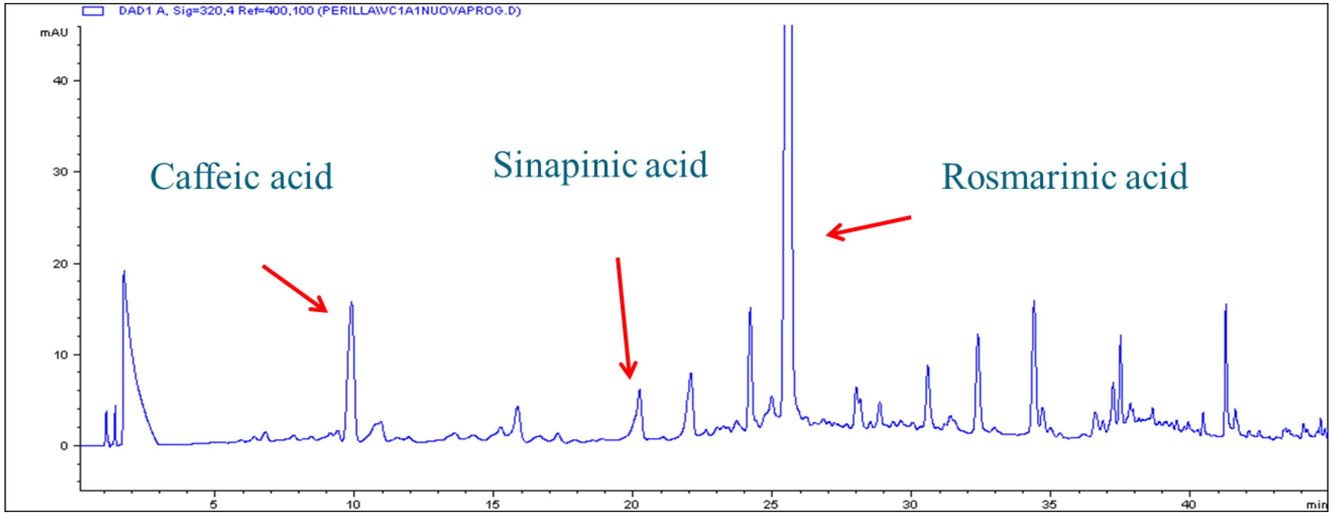

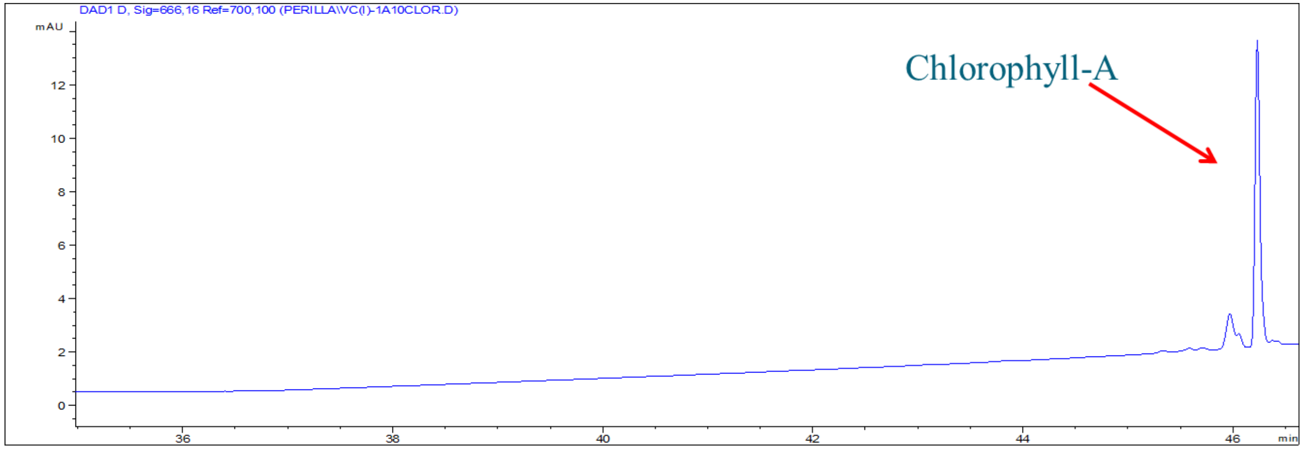

Figure S1. Representative HPLC-DAD chromatogram of polyphenolic (rosmarinic, caffeic and sinapic acids) and chlorophyll a extracts of perilla leaves monitored at 320 nm and 666 nm, respectively.

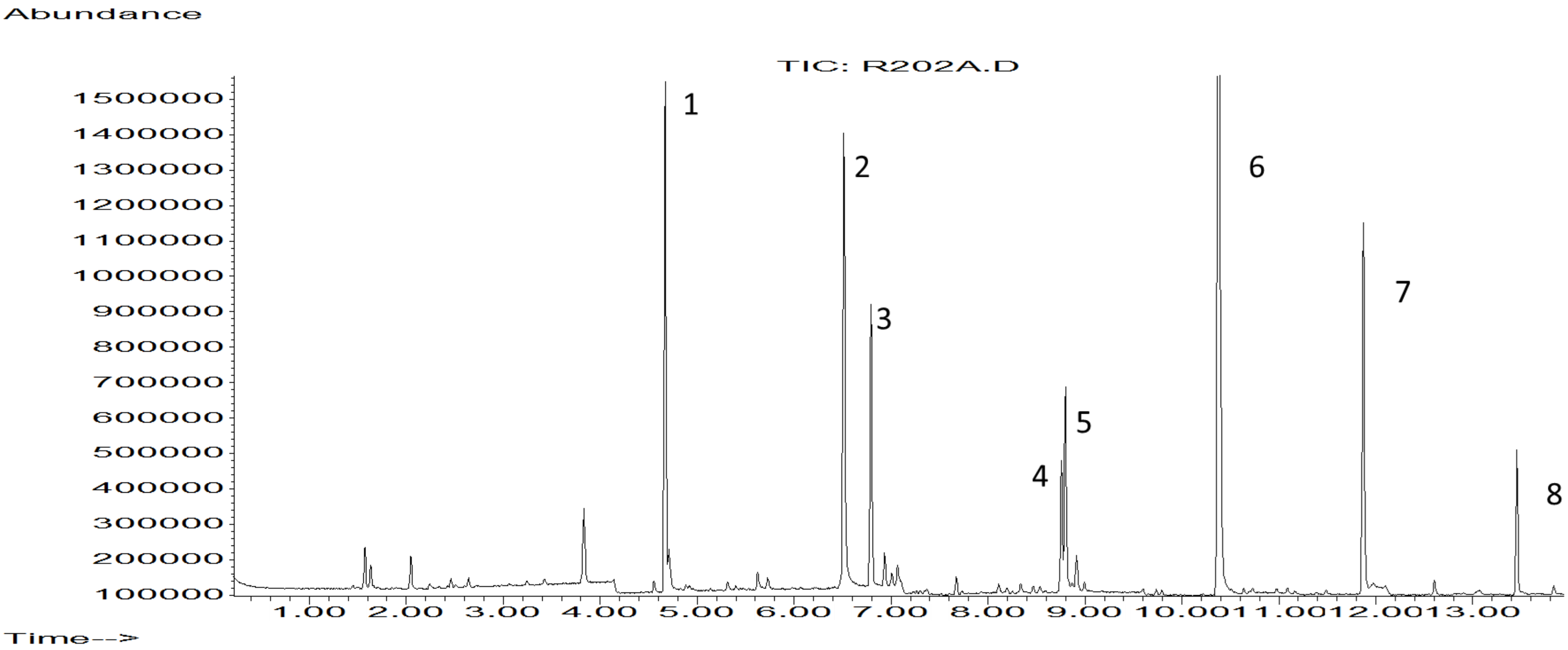

Figure S2. Representative SPME-GC/MS chromatogram of aroma volatile compounds of perilla leaves (1: 2-hexenal, 2: Benz-aldehyde, 3: 1-octen-3-ol, 4: Durenol, 5: - linalool, 6: Perilla aldehyde, 7: Thymoquinone, 8: Caryophyllene).
